# Birds Reveal their Personality when Singing

**DOI:** 10.1371/journal.pone.0002647

**Published:** 2008-07-09

**Authors:** László Zsolt Garamszegi, Marcel Eens, János Török

**Affiliations:** 1 Department of Biology, University of Antwerp, Antwerp, Belgium; 2 Department of Systematic Zoology and Ecology, Eötvös Loránd University, Budapest, Hungary; University of Exeter, United Kingdom

## Abstract

**Background:**

Individual differences in social behaviour may have consequences for mate choice and sexual signalling, because partners should develop preferences for personalities that maximize reproductive output. Here we propose that behavioural traits involved in sexual advertisement may serve as good indicators of personality, which is fundamental for sexual selection to operate on temperament. Bird song has a prominent and well-established role in sexual selection, and it displays considerable variation among individuals with a potentially strong personality component. Therefore, we predicted that features of song would correlate with estimates of personality.

**Methodology/Principal Findings:**

In a field study of free-living male collared flycatchers, *Ficedula albicollis*, we characterised personality based on the exploration of an altered breeding environment, and based on the risk taken when a potential predator was approaching during a simulated territorial interaction. We found that explorative and risk-taker individuals consistently sang at lower song posts than shy individuals in the presence of a human observer. Moreover, males from lower posts established pair-bonds relatively faster than males from higher posts.

**Conclusions/Significance:**

Our results may demonstrate that risk taking during singing correlates with risk taking during aggression and with exploration, thus personality may be manifested in different contexts involving sexual advertisement. These findings are in accordance with the hypothesis that the male's balance between investment in reproduction and risk taking is reflected in sexual displays, and it may be important information for choosy females that seek partners with personality traits enhancing breeding success.

## Introduction

Personality, i.e. the consistency of behavioural responses displayed in different situations, determines how individuals generally cope with challenges in their physical and social environment [1]–[3]. From the evolutionary viewpoint, personality can in general be related to fitness [4], because temporally stable individual variations in a suite of behavioural traits may drive important life-history tradeoffs in humans and in many animals [e.g. 5], [6]–[10]. For example, one response (e.g. aggression) may be beneficial in one context (e.g. social environment), while its correlated response (e.g. exploration) in another context may involve costs (e.g. predation risk) [11], [12]. Moreover, individuals having little future prospects may invest heavily in current reproductive efforts and take high risks in various situations. Hence, risk taking and personality may mediate the trade-off between current and future reproductive success [13].

Personality traits may shape mating decisions, because certain behavioural characteristics indicate individual qualities that affect reproductive output [14]–[16]. In a socially complex environment like in humans, temperament may function as a costly signal of genetic quality, because certain behaviours are difficult to display in a socially attractive way if a high mutation rate impairs cognitive and communicative abilities [17]. Moreover, pair seeking individuals may use clues about the partner's parental abilities, and choose mates with behavioural syndromes that help offspring thrive [17]. In species with social monogamy and biparental care (such as in humans and many passerine birds), individuals can also mate assortatively, because pair members may prefer matching their personalities in a way that promote efficient cooperation between them resulting in mutual reproductive benefits [18], [19].

A key prediction of models of personality-mediated sexual selection is that individuals should be able to assess differences in behavioural norms, and use information about personality in their mating decision. Mate choice generally relies on sexual characters [20], thus it raises the question whether male ornaments can reflect how the bearer behaves in an entire suite of ecological situations and how it copes with challenges in the physical and social environment. Given that the consistency of behavioural responses across contexts lies at the heart of the personality concept [1], [12], [21], behavioural traits, which have themselves a personality axis are the most likely candidates to evolve as signals of personality, because signal reliability can be maintained by correlated behaviours. Here, we suggest that bird song, one of the most important models in the field of sexual selection [22], [23], has several features that make it a suitable indicator of temperament.

There are at least three reasons to predict that features of song are parts of a behavioural syndrome, which could make them as potential signals of personality. First, bird song is an acoustic trait that does not only attract the interest of females but also predators [23]. Therefore singing males expose themselves to predation risk, and their investment in vocal displays should thus indicate how they balance between reproductive investment and predator avoidance [24], [25]. Individuals producing long songs at high rates and at exposed sites will attract predators and will necessarily be risk takers. Hence, only males of high quality can cope with the high costs of elaborate singing due to predators resulting in the linked evolution of costly song traits and risk taking. Second, bird song is a highly plastic trait, and its composition may be shaped by experience and influences from the vocal environment [26], [27]. If personality has consequences for the exploration of novel habitats and breeding dispersal [28], bolder individuals exploiting several environments should encounter with a range of different vocal stimuli, and have a higher chance to incorporate them into their own repertoire than shy individuals. This would lead to individual differences in song repertoires that mirror the degree of contact with the acoustic environment as shaped by personality. Finally, shared physiological regulation can also mediate a link between temperament and song, as individual variations in stress-responsiveness can be manifested across various stressful situations. Accordingly, adaptation for the metabolic stress due to the production of costly songs can affect the levels of stress hormones and proteins [29] that also govern stress behaviours in a risky ecological situation [30], [31].

We therefore investigated the relationship between song performance, individual differences in explorative behaviour and risk taking in male collared flycatchers (*Ficedula albicollis*), which are European, hole-nesting passerines with an elaborate singing behaviour [29], [32]. Previous studies revealed that individual behavioural phenotypes seem consistent over time and across situations in this species, and thus personality may be a potential subject to reproductive and life-history trade-offs. For example, territorial aggression has significant within-individual repeatability [33], and correlated behavioural responses exist across at least three ecological situations [L.Z. Garamszegi, M. Eens, J. Török submitted manuscript]. In a field study in their natural environment, we first characterised behavioural responses of males to a breeding situation, in which a novel object was mounted. This personality test relied on the comparison of the typical nest-box presenting behaviour during female presence between unaltered and altered environmental conditions (i.e. individuals showing similar activity in the familiar and altered environment were considered bold, while those that decreased their activity at the modified nest box were treated as shy). Second, we also assessed risk taking by measuring the distance at which individual males flee from an approaching predator when the focal bird was engaged in a stimulated territorial interaction. Third, we recorded the song of the same males, and estimated song post exposure (position at singing relative to the surrounding vegetation), song rate, song length and repertoire size, and related them to our indexes of boldness and risk taking. Finally, we estimated the relationship between mating success via the speed of pairing and potential signals of personality. Our general prediction was that the song variables with personality component would correlate with exploration rate and fearfulness, and that the same features of song will be related to pairing success.

## Results

**Figure 1:**
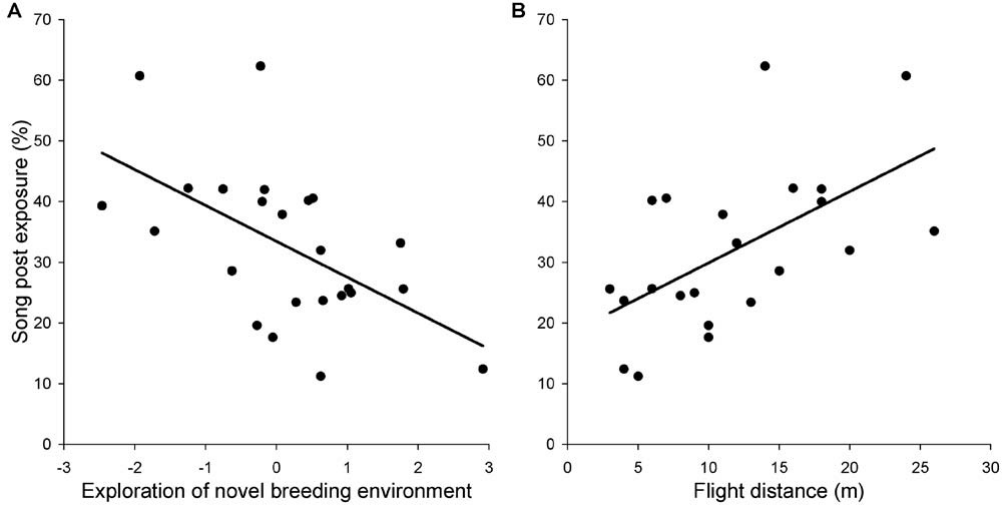
Relationships between personality and song post exposure in male collared flycatchers. Personality was estimated by (A) exploration of a breeding environment that was altered with a novel object and (B) risk taking when a human approaches during a territorial interaction. Positive scores for exploration indicate boldness, while negative values reflect fearfulness toward an altered breeding situation. Flight distance is an inverse estimate of risk taking, with low estimates representing individuals that allow a potential predator to approach them closely. Song post exposure is the average height of singing (in %) relative to the surrounding vegetation.

The observed correlations between song and personality traits are summarised in Table 1. The strongest relationship was found for song post exposure, which indicated that males that sang at relatively low posts were explorative in the altered breeding environment test (Figure 1A). On the other margin, shy males sang at high posts. The analysis of risk taking during territorial activity showed a positive correlation between flight distance and song post exposure (Table 1, Figure 1B) suggesting that risk taking birds that allow a potential predator to approach them closely before fleeing sing at lower song posts on average. The relationship between different personality traits and song post exposure was similar when the effects of arrival date and time of observation were held constant (exploration: r = −0.566, 95% CI = −0.789/−0.216; flight distance: r = 0.649, 95% CI = 0.313/0.841), when we controlled for the co-variation between song variables (exploration: r = −0.422, 95% CI = −0.711/−0.012; flight distance, r = 0.519, 95% CI = 0.125/0.772), and when we factored out local breeding experience (exploration: r = −0.452, 95% CI = −0.759/0.019; flight distance, r = 0.545, 95% CI = −0.054/0.775). Song length also tended to be related to exploration (Table 1), but such an apparent relationship might be mediated by an outlier caused by a male singing extremely long songs (non-parametric test that relies on the ranking of individuals provides weaker effects: r = 0.280, 95% CI = −0.139/0.614), and by a correlation between song post exposure and song duration (r = −0.448, 95% CI = −0.721/−0.054).

**Table 1:** Relationships between features of song and the degree of exploration of an environment altered with a novel object and flight distance reflecting personality in male collared flycatchers.

Exploration	Effect size correlations	95% CI: lower CI/upper CI	P
Song length	0.432	0.035/0.711	0.035
Song post exposure	−0.563	−0.788/−0.207	0.004*
Song rate	−0.176	−0.541/0.245	0.410
Song complexity	−0.288	−0.619/0.131	0.172
Repertoire size	−0.177	−0.549/0.254	0.420
**Risk taking**			
Song length	−0.210	−0.580/0.232	0.349
Song post exposure	0.582	0.213/0.806	0.004*
Song rate	0.139	−0.300/0.530	0.536
Song complexity	0.309	−0.129/0.646	0.161
Repertoire size	0.266	−0.175/0.618	0.231

Exploration was estimated based on the nest presentation behaviour of males at a modified nest box, which was expressed relative to presentation behaviour at the natural nest box. Flight distance is an inverse estimate of risk taking, as it reflects the distance to which a potential predator can approach the individual when the male is engaged in a territorial fight. Correlations (Pearson's *r*) together with the associated 95% confidence intervals are presented to reflect the strength of relationships in the form of effect size. For demonstrative purposes significance levels are also given (^*^: significant after Bonferroni adjustment for ten tests, i.e. P<0.005). N = 24 for each test in relation to exploration, except for repertoire size, as song recordings for one male provided less than 20 songs making it impossible to estimate repertoire size according to our standard; N = 22 for tests of risk taking.

**Figure 2:**
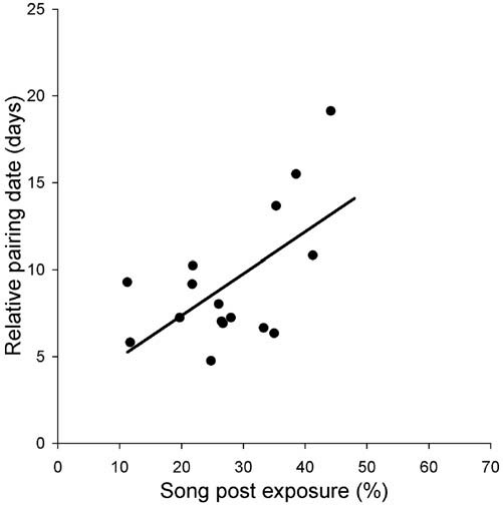
Relationships between song post exposure and pairing success. Pairing success was measured by relative pairing date. Birds pairing relatively late are those that demonstrate longer intervals (in days) between arrival and the start of egg laying than birds realizing quick pairing. Data are residuals from a multiple regression, in which the date of arrival was included to control for date effects.

To test whether songs from lower posts are perceived as more attractive than songs from higher posts, we estimated the correlation between song post exposure and mating success. We found that males singing from lower posts established pair bonds faster than others using higher posts (r = 0.564, 95% CI = 0.095/0.828, when controlling for date effects; Figure 2).

## Discussion

Here we found that males of the collared flycatcher display systematic differences in song post exposure with some individuals showing preferences for higher song posts while others for posts closer to the ground. This variation has been related to exploration and risk taking, as singers from lower elevations also take higher risk in the presence of a potential predator and explore an altered breeding environment more boldly than singers from higher elevations. Moreover, song post exposure may determine mating success, as males advertising from lower posts paired more quickly. These findings are in accordance with the hypothesis that individual differences in the choice for singing site reflect individual differences in personality in flycatcher males, and song post exposure may be a subject to sexual selection. On the contrary, we did not find strong evidence for other song traits having evolved as signals of personality.

We proposed three mechanisms that can drive personality to be involved in sexual advertisement via bird song. First, the cost of songs due to predation may favour risk-taking to evolve as a component of trait elaboration. Accordingly, if song post exposure is shaped by predictable predator pressure, we should expect risk-averse males to consistently choose predator safe microhabitats for singing. We do not have information about the height-dependent predation risk in our study site, thus we can only speculate that a complex forested habitat may follow a heterogeneous predator pressure along the vertical axis. For example, displaying birds may generally expect different predators from the canopy and from the ground [25], or they may adjust song post exposure to the concealment perceived at different sites [24]. Therefore, selection pressures that vary perpendicularly may constrain individuals to generally apply consistent micro-habitat choice for singing depending on the risk they take.

It is also plausible that the detected patterns are specific to the experimental conditions, in which an observer making song recordings was unavoidably in the vicinity of the nest box to obtain recordings of good quality. In this case, it is not necessary to assume a general preference for specific song posts and a systematic predator pressure along the vertical axis. Hence, realized songs posts can reflect risk taking during singing but only when a human observer is present on the ground. However, we demonstrated that such risk taking during sexual advertisement correlates with risk taking in another context (distance at which a potential predator can approach during aggression) and with exploration. This finding still implies that personality can be manifested in sexual displays, for which we could not locate any previous evidence. Our results may thus suggest that the presence of an observer making song recordings in the territory can trigger directly a trade-off adjustment from the males, in which they balance the immediate benefits and costs of vocal advertisement close to the nest box. Such a flexible choice for song post can cause risk-averse individuals to recede to sing in the safe canopy level, but only when a potential predator is present. However, if the relationship between song post exposure and personality applies to the experimental situation only, it still has a consequence for sexual selection. This is because singing sites exploited during the presence of a human observer, or its correlate thereof, can predict relative pairing date indicating that the measured trait may be important for mating success.

The second mechanism that we considered links personality to bird song through exploration, as bold individuals may have more opportunity to confront with different habitats and explore a diverse acoustic environment than shy individuals, which could have consequences for the composition of repertoires. We failed to find a strong association between repertoire size/strophe complexity and components of personality indicating that the number of unique vocal elements in the repertoire (figures) is unlikely to reflect personality. However, it remains a testable prediction that explorative males have higher chance to encounter and learn rare elements that are sung by few individuals in the population than less explorative conspecifics. This would imply compositional differences between individuals that are independent of repertoire size.

The third mechanism suggested operates via shared physiological regulation between personality and song. Our previous study has revealed that the production of longer songs is accompanied by higher levels of heat shock proteins, which may indicate that individuals cope by physiological means with the cost of singing [29]. Song duration is also associated with song post exposure, and there was also some tendency for this trait to be related to exploration (Table 1). We did not estimate song post exposure in the previous physiological study, but based on the correlations observed between metabolic and song traits, we cannot exclude the possibility that there is a relationship between the levels of heat shock proteins and song post. If this association were discovered, this could indicate that individuals singing at exposed sites cope with the increased risk they take by elevated levels of stress proteins. Stress hormones, such as corticosterone is known to serve as the physiological basis for individual differences in boldness [30], [31]. Further studies are needed to elucidate the role of the same hormones in mediating singing behaviour.

Good genes models of sexual selection are in accordance with the finding that song post is related to an estimate of risk taking, if individual quality is associated with the risk taken during singing. However, alternative mechanisms should also be considered. For example, breeding experience may be a confounding factor, as individuals unfamiliar with the study site are adapted to breed in natural cavities that are likely found at higher canopy levels. Hence, risk-takers may be experienced local birds that have learnt to exploit the space around the nest boxes, while immigrant or inexperienced birds may tend to remain at higher levels during singing. Our data indicates that this mechanism mediated by breeding experience is unlikely, because the relationship between risk-taking and song post was not affected by breeding experience. Moreover, we also assessed the role of territory quality, because males occupying nest boxes of superior quality may take higher risk and prefer advertising close to these nest sites. However, we can exclude this possibility as well, as song post remained associated with personality traits when the date of arrival was held constant. Arrival order of males translates to differences in territory quality, as early arrival is generally known to lead to the occupation of prime breeding sites [34].

We also found that song post exposure was related to pairing success. This is consistent with the hypothesis that females favour risk taker males that produce songs from exposed sites (in general, or in the specific experimental situation in particular). Listeners of songs might discriminatively respond to males that produce songs in different sites, which may have consequences for mating success. Each micro-habitat has particular transmission properties [35], and vocal signals emitted from predator-safe sites may be unsuitable for production of attractive signals. For example, there may be certain positions in the forest, from which listening females are more likely to hear and respond to songs from the ground levels that sound louder than songs from the canopy level. Moreover, a female visiting a territorial male may inspect singing behaviour for a longer period [36], during which she could have a choice to directly assess the preferred song posts of the singer.

Female preference for signals of bold personality may convey several fitness advantages. In line with the costly signalling theory of sexual selection, males that bear the costs of singing at unsafe posts are likely of superior quality and harbour good genes for the offspring [17]. Moreover, exploratory males can successfully exploit alternative habitat patches and deliver variable food with rich nutrition value [37]. Personality may also be related to territory quality, as boldness and exploratory behaviour positively correlate with aggression that can be translated into competitive success in territory settlements [18], [38]. Alternatively, risk-taking males may have high commitment in the current breeding attempt, because individuals having little prospects for future may be expected to invest a lot in current efforts and thus should take high risks in various situations [13].

Our results have important implications for both the evolution of sexual characters and temperament traits. One of the key assumptions of theories of sexual selection is that the signal reliability of male traits is maintained by bearing costs due to predators [20]. Surprisingly, there is little evidence of predation affecting the design of vocal signals in birds [39], [40]. Here we demonstrated for the first time that individual decisions about investment in sexual signalling and predator avoidance inherently involve personality components. Given that risk taking is likely to mediate reproductive trade-offs, models of sexual selection could be better understood in the light of the personality concept. Finally, our findings add to our knowledge about the evolution of personality. A recent theory proposed that temperament may be used as a sexual signal of genetic quality in humans [17]. However, the relationship between male displays and personality traits may be more general and apply to other animals.

## Materials and Methods

The collared flycatcher is a small migratory and hole-nesting passerine in Central European broad-leaved forests, which has a socially monogamous mating system with facultative polygyny [41]. After arriving from the wintering sites, males preferably occupy artificial nest boxes for breeding, but may occasionally establish territories around natural cavities [42]. These breeding territories are advertised by active displays, which involve singing and typical nest presentation behaviour. Males defend their prospective breeding sites by aggressive interactions against intruding individuals. Females choose among advertising males, then build nests alone and lay and incubate 6–7 eggs. Both sexes provide parental care at the nestling stage. By placing nest boxes about 1.5–2 m above the ground, we established breeding plots at Pilis Field Station near Budapest (47°43′N, 19°01′E), Hungary in 1981 for the long-term study of the species [see 43]. Fieldwork for the current study was carried out during 2007, when we recorded explorative, risk taking and singing behaviour of unpaired males during the most active morning singing period (usually between 05.00 and 10.00h).

To assess exploration, we characterised the behaviour toward an experimentally altered breeding environment relative to a familiar breeding environment. To do so, we first monitored the natural nest-presentation (courtship) behaviour of resident males, which were stimulated by a decoy female presented at the occupied nest box. We placed the caged female on the top of the nest box, from where females usually observe nest-presenting males in a natural situation. Nest-presenting males frequently approach the nest box and call the female to enter the nest box and to evaluate its quality. These approaches can be characterised by excited flights around the nest box and repeated landings on its top and entrance hole. Behaviours displayed during the simulated female-visit were used to describe general activity at the familiar breeding site (control activity). Then, we modified the breeding environment by attaching a white paper sheet below the entrance hole of the nest-box, and measured the same behavioural variables by using the same female stimuli (experimental activity). To avoid habituation, we left at least half an hour between the two behavioural observations, during which the male became engaged in vocal advertisement, and we could record its song (see below). We measured three traits in both the control and experimental situations during a 10-minutes of observation each. We counted the sum of the number of landings on the top of the female cage, nest box and entrance hole; measured the duration of time (in sec) that the male spent on the cage, entrance hole or within the box altogether; and the latency (in sec) elapsed between the appearance of the resident male on its territory (i.e. the detection of female as indicated by the typical nest box presentation behaviour) and the first landing on the entrance hole of the nest-box. These measures were strongly inter-correlated (r>0.55), thus we achieved dimension reduction by a principal component analysis that produced a single axis to describe the male's interest towards the control and the experimental breeding situation, separately (explained variance in the three measured behaviours: control situation, 77% experimental situation, 79%). We expressed exploration as the experimental activity relative to the control activity by calculating residuals from a linear regression between the two principal components summarising nest presentation in different situations (correlation between behavioural principal components across the control and experimental situation: r = 0.805, N = 26, P<0.001). Hereafter we used these residuals to reflect the degree of response to the altered environmental situation that is independent of activity, with high values indicating more explorative behaviours in the altered breeding opportunity than lower values. The behavioural variables during nest box presentation (both control and experimental situations) were recorded from a distance of c.a. 25–30 m by the same observer (LZG). We measured exploration for N = 24 males that were also recorded for their songs.

The distance at which an individual flees from a potential predator represents a measure of risk-taking [25], [44], [45]. To estimate personality via risk taking, we adopted a simple technique to assess risk taking when a potential predator (human) is approaching. We simulated a territorial challenge by exposing males to a male intruder that was presented in a small wire cage placed 2–3 m from the nest box (c.a. 0.5 m above the ground level). When the resident had been located on the cage while showing clear intention to fight as a component of typical territorial defence [33], the observer (LZG) moved at a normal walking speed towards the focal male (from a distance of c.a. 25–30 m). When the individual took flight from the cage because of the human disturbance, the distance between the position of the observer and the experimental cage was measured as the number of steps (which approximately equals meters). This distance was recorded as the flight distance. Low numbers for this variable mirror high risk taking, because birds with short flight distance allow potential predators to approach them closely. We could estimate risk taking for N = 22 individuals with information on song.

We made an effort to use different stimulus birds across different tests, as the identity and attributes of presented males and females may be important [see 33], [46]. Hence, when characterising exploration, we utilized 17 different females randomly (each female was used 1.52 (±0.25 s.e.) times on average). In tests of risk taking, we used 10 males (each male was used 2.5 (±0.45 s.e.) times on average). We exchanged stimulus birds, as they became available from our parallel capturing sessions at a distant breeding plot. Therefore, we assumed that the focal birds were unfamiliar with the stimulus birds.

Song was recorded after the presentation of a female stimulus by using a Sony TCD-D8 DAT tape recorder and a MD 21 U microphone [29], [32], [47]. To obtain song recordings of sufficient quality, the observer (LZG) was positioned closer to the nest box (c.a. 10 m) than in the previous behavioural tests. This distance was determined based on previous experience [29], [32], [47], which suggested that focal birds perform apparently undisturbed singing behaviours when recorded under these conditions. We recorded each male for at least 10 minutes to obtain a sufficient number of songs with minimal background noise. We estimated song rate as the number of songs/min based on the available recordings. We selected 20 consecutive songs of good recording quality and analyzed them spectrographically. We measured song length (in seconds) and complexity (no. unique elements/no. elements within song) for each song: Given the repeatability of these song traits within individuals [32], we averaged these variables for each male. Total figure repertoire size was estimated by the number of unique figures detected in the entire sample of 20 strophes. Song post exposure was coded during song recording, when for each song, we determined the height of the birds at singing relative to the surrounding vegetation. Song post exposure was thus expressed as the percentage of all available song posts, ranging from 0 % when singing occurred on the ground to 100 % when the male sang on the top of the canopy [25], [48]. Within males, the variation between songs was consistent, which could be described by a significant repeatability index (arcsine-square-root transformed data: R = 0.478, F_23,567_ = 11.689, P<0.001). This estimate was comparable with other song traits, such as song rate or song duration [e.g. 32], [49], [50].

We estimated pairing success of males by estimating relative pairing date, which is the number of days elapsed from arrival (date of observation) until the start of egg laying (N = 16 males were re-found breeding). Relative pairing date is generally used to reflect how quickly a male finds a partner, as quick and early pairing is likely to be caused by female preference and successful mate attraction [51]–[56]. Arrival date may bias pairing date, because early arriving, high quality males should wait longer to pair than late arriving poor quality males, as early in the season there may be few females available. To control for the confounding effect of arrival date, we included arrival date in the analyses, where relative pairing date was used.

We also used information for breeding experience, as in theory, it may confound the relationship in focus (see Discussion). Breeding experience was estimated by the number of breeding attempts as detected in our long-term survey prior 2007. We could locate this information for N = 18 males. Young (1^st^ year breeders) birds and males that had not been captured earlier were assigned as inexperienced breeders and were considered to have no previous breeding attempt in our nest box plots. The date of first observation (the date of behavioural tests and song recordings) was used to reflect arrival date, which was also taken into account as a control variable. We assumed that the date of observation is a good surrogate of real arrival date, because we monitor our breeding plots for newly arrived, displaying birds in a standard way. It is therefore likely that males used in this study were recorded just upon their arrival.

For the statistical analyses, we checked the distribution of variables, and applied statistical transformations if necessary to meet the parametric criteria. To determine the strength and direction of the relationship between the investigated traits, we calculated Pearson's r correlations. When confounding variables were considered (such as date and time effects, or breeding experience), we calculated partial correlations between the traits of interest. Multiple testing involves the risk of committing type I errors [57]. Therefore, for the interpretation of the correlations between song traits and risk taking, we present effect sizes with the associated 95 % confidence intervals (CI) for each particular relationship [58]. However, this approach is not yet widely applied, thus for demonstrative purposes we present significance levels for each relationship, and consider Bonferroni adjustments for multiple tests.
